# Roost sites of chimney swift (*Chaetura pelagica*) form large‐scale spatial networks

**DOI:** 10.1002/ece3.7235

**Published:** 2021-03-20

**Authors:** Courtney E. le Roux, Joseph J. Nocera

**Affiliations:** ^1^ University of New Brunswick Fredericton NB Canada

**Keywords:** aerial insectivore, chimney swift, roost, roost network, social network analysis

## Abstract

Several biodiversity‐centered metrics exist to quantify the importance of landscape and habitat features for conservation efforts. However, for species whose habitat use is not quantified by these metrics, such as those in urban areas, we need a method to best identify features for targeted conservation efforts. We investigated the use of social network analysis (SNA) to identify and quantify these critical habitat features. We used SNA to identify network existence in chimney swift (*Chaetura pelagica*) roost usage, quantify the importance of each roost site, and evaluate the impact of the loss of key sites. We identified a network consisting of ten chimney swift roosts in southern Nova Scotia, Canada, and found that 76% of swifts used more than one roost throughout the breeding season. We also isolated three key (most connected) roost sites. We evaluated the effect of loss of these key sites on the network by using a Wilcoxon‐Pratt signed‐rank test and by analyzing the structure of the subsequent network. We found that connections between roosts and the structure of the network were significantly affected by the loss of these key sites. Our results show that SNA is a valuable tool that can identify key sites for targeted conservation efforts for species that may not be included in conservation efforts focused purely on biodiversity.

## Graphical abstract

Social network analysis (SNA) reveals that roost sites separated by hundreds of kilometers form a spatial network for Chimney Swifts, a species long believed to have fidelity to single roosts. SNA has never been applied to the spatial ecology of wildlife in this way and promises to aid in identifying sites in greatest need of conservation.

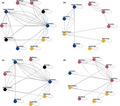

## 1 INTRODUCTION

Habitat loss is a primary driver of species population declines (Brown & Paxton, 2009; Schmiegelow & Mönkkönen, 2002; Stuart et al., 2004), and many conservation efforts are focused on the identification, restoration, and protection of important habitats or landscape features to combat the impacts of these losses. Despite these efforts, landscapes continue to change, and important wildlife habitats continue to be lost. Not only has space become a limiting factor for some species’ survival, but resources for efforts to conserve the habitats that remain are also limited. To use these resources effectively, it is imperative to know which locations or features are most important for maintaining species’ populations. This approach can be seen in attempts to conserve areas based on the number of endemic species (Davis et al., 2008; Lei et al., 2003; Myers et al., 2000), total species diversity (Song et al., 2016), rankings of biodiversity (Sarkar et al., 2006), and habitats associated with species with high likelihood of persisting (Williams & Araujo, 2000; Yirka et al., 2018). Social network analysis is an emerging method to identify key sites for a single species based on its importance in maintaining connectivity among a network of landscape features. A network analysis approach can provide guidance for the conservation of sites for species that may not be included in biodiversity‐focused metrics.

Network analysis is a developing analytical technique used to define and quantify “meaningful” features in the context of landscape or habitat features that form interactive networks. The ability of each feature to maintain connectivity of the larger network, or to connect remote areas, are examples of quantifying meaningfulness. Given this, we sought to test the hypothesis that social network analysis can be used to identify key features within a network of landscape features for targeted conservation of a single species at risk: the chimney swift (*Chaetura pelagica*; classified as threatened in Canada [COSEWIC, 2018]).

Social network analysis (SNA) allows the investigation of social structures based on the mathematical concept of graph theory (Pavlopoulos et al., 2011). These graphs are composed of individuals or features of interest (nodes) and connection or movement between them (links or edges). Using SNA, it is possible to identify which nodes are important for maintaining movement within the network (centrality), and the degree to which each node is connected to all others (node degree). A primary assumption of SNA is that the number of connections to a node indicates its importance to the network. SNA can also identify which nodes are most important for maintaining movement between these communities (betweenness), and which subsets of nodes are more linked to each other than to other nodes in the network (communities). The ability to identify communities within a network could have management implications. A well‐connected community may not benefit by the addition of a new roost structure as much as a small or weakly connected community.

Social network analysis is often used to study human interactions, including in advertising (Brown et al., 2007; Kempe et al., 2003), determining friendships (Eagle et al., 2009), corporate and business structure (Reagans & Zuckerman, 2001; Tsai, 2002), and political associations (Yang et al., 2012). Use of SNA in ecological research has been steadily increasing and has been applied to how animals interact with others within their group (Kasper & Voelkl, 2009; McCowan et al., 2008; Sueur et al., 2011). These studies tend to categorize individuals as the nodes or focal points of the graphs, and associations between individuals as the links.

Few ecological studies have examined how features of the environment act as nodes. The use of roosts as nodes by bats has been evaluated using SNA in terms of spatial distribution of roost trees (Johnson et al., 2012; Rhodes et al., 2006), influence of resources on network structure (Chaverri, 2010), and how nodes impact the spread of disease (Fortuna et al., 2009). Other studies have considered habitat patches to be nodes in a network and used SNA to evaluate connectivity between these patches (Baranyi et al., 2011; Calder et al., 2015; Rubio & Saura, 2012). A benefit to this approach versus only traditional mapping is the ability to evaluate the impact of node loss on the network (Calder et al., 2015; Mourier et al., 2017).

We used a social network analysis of the movement of radio‐tagged chimney swifts between roost sites to (a) investigate whether roosts formed a large‐scale network that was used by multiple swifts during the breeding season, (b) to identify the most significant roost chimneys within the network, and to (c) predict the outcome of the loss of one of the most significant roosts. We hypothesized that the roost chimney with the greatest number of connections would be the most significant, and that its loss would dismantle the roost network. This information could be used to guide conservation efforts by providing a quantified ranking of the ecological importance of each feature within the network. We chose chimney swifts (hereafter: swifts) as a model species because they are highly aerial and, as a result, they physically interact primarily with their roosts (nodes) and nest sites, and no other landscape features. In this way, roost chimneys act as islands and provide a unique and simplified network to test the applicability of SNA in this environment.

Roost sites are fundamental for swift ecology, offering protection from predation, providing more stable microclimates, and protection from the elements (Steeves et al., 2014). Roost sites are especially important in poor weather, providing a protected space for groups of swifts to rest, conserve heat (COSEWIC, 2018), and reduce water loss (Farquhar et al., 2018) during suboptimal foraging conditions associated with poor weather. Not only do swifts use these structures throughout the energetically expensive migrations, but also throughout the breeding season (COSEWIC, 2018; Steeves et al., 2014) when they are used by nonbreeders, failed breeders, and the nonincubating member of a successfully breeding pair (COSEWIC, 2018). These roosts are rare across the landscape because they are typically large masonry chimneys, structures which are becoming obsolete. This makes them important features for conservation efforts. By using SNA to determine their connectivity and quantify their significance to the overall network, a targeted conservation approach could be considered for roost site preservation.

## 2 MATERIAL AND METHODS

### 2.1 Study species

Chimney swifts provided a model system for evaluating the applicability of SNA in this context. Swifts are small aerial insectivores that breed in eastern North America and overwinter in the Amazon basin. They have been experiencing drastic population declines of −4.9% per year since 1970 (COSEWIC, 2018), which is hypothesized to be due to a complex combination of factors that include a loss of nesting and roosting habitats (Fitzgerald et al., 2014). The likelihood that the loss of these important sites is leading to widespread population declines highlights the importance of finding a way to identify which sites should be the focus of conservation efforts given limited conservation resources. It is estimated that only 60% of breeding age adults reproduce per year, leaving a large proportion of the population that will continue to roost communally throughout the summer. It has been assumed that these roosting swifts show a high degree of roost site fidelity, but this has not been explicitly tested and may not be the case. If swifts do change roost sites within the breeding season, then these sites could form an interactive network across the landscape and the use of SNA for identifying key sites of conservation value.

We expected roost sites to form an interactive network over a large landscape due to swifts’ ease of movement while foraging and the limited number of roosts available. The ability to forage over a large area and roost in a different location would allow swifts to acquire greater food resources while still obtaining the benefits of communal roosting. This may be of particular value during certain weather patterns or periods during the summer when energetic expenses may exceed the available prey resources in an area.

### 2.2 Site selection

We captured and tagged 53 swifts at the Caledonia roost in Kempt County, Nova Scotia, Canada (44.4181°N, −65.0546°W) throughout June of 2018 and 2019. This roost is of moderate size (1.2 m^2^, 5 m tall), hosting up to 700 swifts during migration, and ca. 250 throughout the breeding season. This masonry chimney is attached to a vacant building that served as a blacksmith workshop in the 1930s to 1960s. The chimney has a partial metal cap with a 0.5 m diameter circular opening in the center. We selected this site for tagging of swifts due to its accessible height that allowed for rapid removal of the trapping device described below. We attempted to reduce this tagging bias by only including birds that left and then returned to Caledonia as representing a link for this roost.

### 2.3 Trapping

Throughout June 2018 (*n* = 20) and 2019 (*n* = 33), swifts were captured using a modified hoop net (Colvin & Hegdal, 1986; Wheeler, 2012) under Animal Use Protocol #18103 (University of New Brunswick). This design consisted of a 55 cm diameter circular frame from which a 1 m long cylinder made from mist net material (38 mm mesh), tapered to a diameter of 15 cm was suspended. The mist net was weighted at the bottom to prevent tangling. The net was suspended in the chimney with the circular frame held in place by a 10 cm rim protruding from the chimney opening.

We lowered the net into the chimney 30 min prior to dusk, before swifts began to enter the roost. The net was removed immediately upon entry of no more than five birds into the chimney, captured birds were extracted from the net, and placed in cotton draw‐string bird bags to be processed one at a time. Capture and transmitter attachment took place for two consecutive nights each week (*n* = 10/week) until all available tags were deployed. By staggering tag deployment, we aimed to reduce disturbance at the roost and minimize the risk of swifts abandoning the site. The staggered deployment also provided a wider date range of data due to limited tag battery life (see below).

### 2.4 Transmitter attachment

We processed birds on the ground, 20 m away from the roosting structure. Handling time was kept below five minutes per individual once removed from the bird bag, and less than thirty minutes after capture. Swifts were fitted with Lotek Wireless nanotags, model NTQB‐3‐2, weighing 0.62 g. These tags measured 19.6 cm (approximately 1 cm of tag, and 18.6 cm antenna), were programmed with a burst interval of 13 s, and had an estimated battery lifespan of ca. 224 days. In 2018, these tags were programmed with unique frequencies for detection with handheld Lotek receivers (SRX800) and coded for the Motus Wildlife Network (Motus; Taylor et al., 2017) in 2019. Swifts were released from the hand 50 m from the roost chimney after processing to reduce disturbance to other swifts that had entered the chimney during processing. They responded to release by flying several circles in the area before entering the roost chimney.

Lotek Wireless nanotags were attached using a modified figure‐8 harness as described by Rappole and Tipton (1991), Doerr and Doerr (2002), and Haramis and Kearns (2000). Due to the high energetic demand swifts face, it was important that the nanotags not remain attached to the swifts indefinitely. To prevent this, harnesses were constructed using 0.5 mm absorbable surgical suture (Vicryl PGA suture, Ethicon, USP 1); we chose this diameter to prevent chafing of the individuals (Doerr & Doerr, 2002; Woolnough et al., 2004). When exposed to the environment, the absorbable suture is expected to dissolve after ca. three months (Doerr & Doerr, 2002) and relieve the swifts of the extra energetic burden of the transmitter. In addition to using absorbable surgical suture, another modification to the Rappole and Tipton (1991) harness design was that we preconstructed the harness with adjustable leg loops, which reduced handling time and provided a secure and customized fit to each bird (Doerr & Doerr, 2002; Streby et al., 2015), decreasing the likelihood of the harness slipping off due to the short and posteriorly positioned thighs of the swifts.

### 2.5 Movement data

**FIGURE 1:**
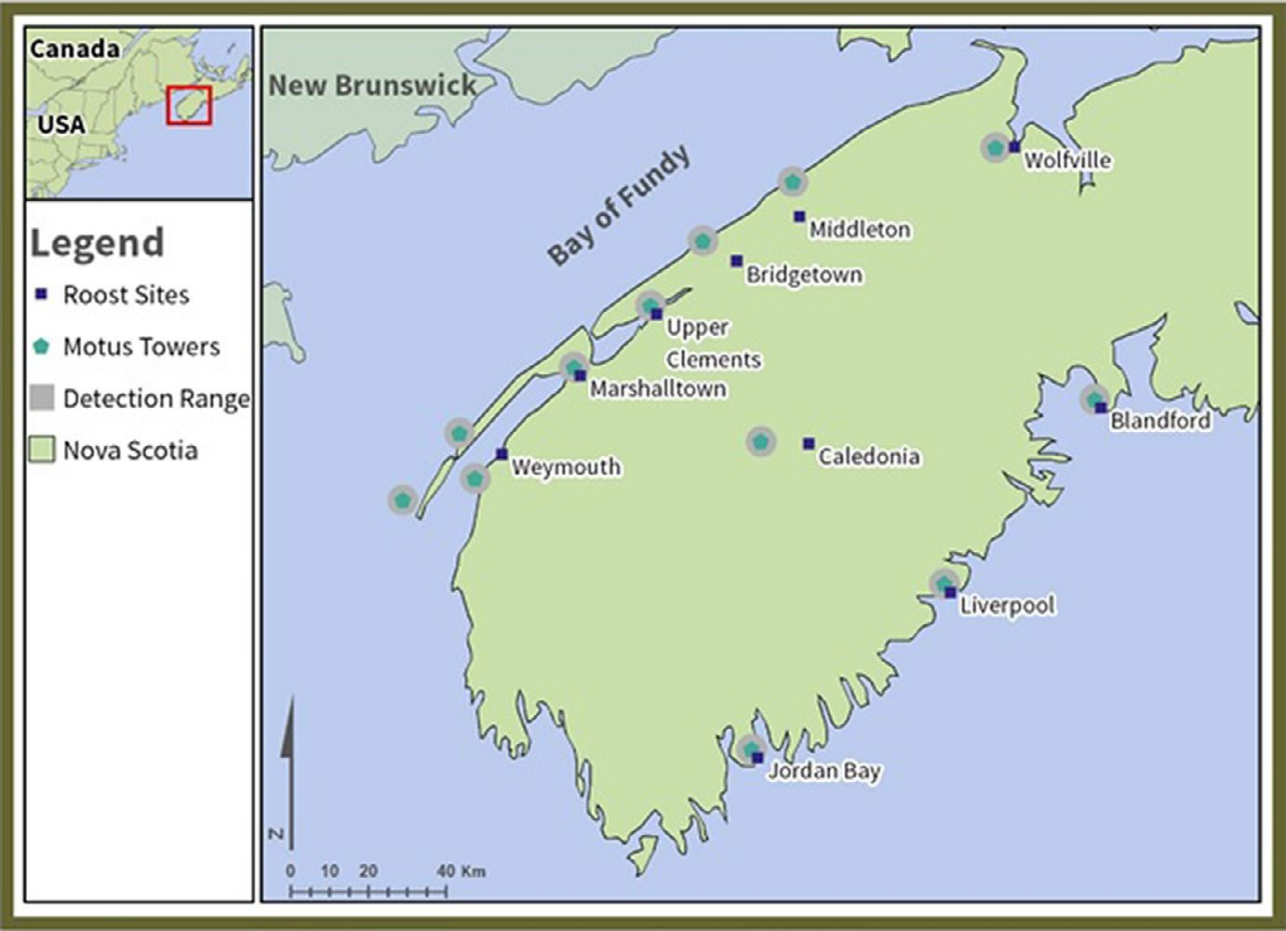
Location of active Motus automated telemetry towers, their detection ranges (Taylor et al., 2017), and chimney swift (*Chaetura pelagica*) roosting sites throughout southern Nova Scotia, Canada, as of September 2019

From June to August 2018, we recorded swifts at roost sites using a handheld Lotek SRX800 radio telemetry receiver with a four element Yagi antenna. We travelled between known roosts once swifts had entered roosts after dusk, scanning at each location for all tagged individuals. We alternated scanning the western and eastern shores of Nova Scotia. For the western shore, we proceeded southwesterly from Wolfville to Weymouth, before progressing inland to Caledonia. Along this route, we scanned at each known swift roost site (Figure 1). The eastern shore route consisted of traveling northeasterly from Yarmouth to Bridgewater, where there were no known swift roosts. We scanned along a 1 km grid after dusk in towns along this route.

This method proved laborious and yielded no detections along the eastern shore, so we used the fixed antennas of the Motus Wildlife Network (hereafter: Motus) in 2019. Motus is a system of automated telemetry receivers that record the location of tagged individuals within range (ca. 4 km) in five‐minute intervals (Taylor et al., 2017). The masonry of the chimney reduces this detectability, so we assumed that a roost site was in the vicinity of late evening and early morning detections. Each Motus tower uses the same single scanning frequency (166.380 MHz), increasing detectability compared to an approach of cycling through frequencies (Taylor et al., 2017). We registered our nanotags with Motus, and upon detecting a signal, the nanotag information was compared to the registration, and raw data were made available via an online server (motus.org).

There were eight known and active chimney swift roost sites within the southern Nova Scotia study site, and 27 active Motus towers (motus.org). Of these roosts, one (Wolfville) was within range of a Motus tower, with the remaining roosts <12 km from active Motus towers (Figure 1). One roost site (Weymouth) was <10 km from two Motus towers and <20 km from another, so data from these three Motus towers were pooled. We assumed that each Motus tower represented data on the nearest roost for the other seven. We identified a chimney as roosting and not nesting based on the frequency of use. We assumed a nesting chimney would record swift presence continuously over the nesting period while a roosting chimney would only record presence over night and on a less continuous basis as tagged swifts moved between roost sites. It is also known that multiple breeding pairs will not occupy the same chimney (COSEWIC, 2018); as such detections of more than one tagged individual at a site would indicate a communal roost as opposed to a nesting site.

### 2.6 Analyses

We filtered detection data to include only the province of Nova Scotia, Canada. Run length is the number of consecutive detections of a single tag by a Motus tower, and short run lengths (≤3) are considered false positives (Crewe et al., 2018). We excluded these detections, as well as all detections outside an 18 hr00–10 hr00 time window, to limit the possibility of detecting birds that were not roosting in the area but merely foraging. Detections after 1 September 2019 were excluded from all analyses, as these were more likely to represent migration movements.

We used the igraph package in R (Csardi & Nepusz, 2006; R Core Team, 2017) with *α* = 0.05 to determine the significance of important roosts, by quantifying degree and closeness centrality, on the structure of the roost network. Tagging all swifts for this study at one site (Caledonia) could overrepresent the importance of this roost to the network. To minimize this risk, only birds that left and then returned to Caledonia were considered a link for the roost (Table 1).

**TABLE 1:** Simplified movement of chimney swifts (*Chaetura pelagica*) among 10 roost sites (five confirmed and five unconfirmed) within southern Nova Scotia

Tag	Roost 1	Roost 2	Roost 3	Roost 4	Roost 5	Roost 6	Roost 7
247	Caledonia	Bridgetown					
250	Caledonia	MIddleton	Bridgetown				
255	Caledonia	Caledonia					
256	Caledonia	Bridgetown	MIddleton	Bridgetown			
257	Caledonia	Caledonia					
258	Caledonia	Wolfville	Bridgetown	Caledonia	Bridgetown		
260	Caledonia	Caledonia					
264	Caledonia	Bridgetown	Caledonia				
265	Caledonia	MIddleton	Bridgetown				
624	Caledonia	Marshalltown	Upper Clements	Caledonia			
626	Caledonia	Caledonia					
628	Caledonia	Upper Clements	Caledonia	Blandford	Upper Clements	Caledonia	Weymouth
629	Caledonia	Caledonia					
631	Caledonia	Upper Clements					
637	Caledonia	Upper Clements					
640	Caledonia	Liverpool	Upper Clements				
641	Caledonia	Marshalltown	Weymouth				
643	Caledonia	Upper Clements	Caledonia	Wolfville	Marshalltown		
644	Caledonia	Upper Clements	Marshalltown	Upper Clements			
645	Caledonia	Jordan Bay	Weymouth				
650	Caledonia	Weymouth	Caledonia				

Note

The tagging site, Caledonia, was only included in social network analyses when a swift either remained in or left and returned to Caledonia.

We identified communities within the network by grouping roosts that shared more connections with each other than they did with the rest of the network (Radicchi et al., 2004). We then used degree and closeness centrality to quantify the importance of each individual roost to the network. Degree centrality is the most basic representation of the importance of a node to a network. It is a local measure, meaning it only considers the node in question, and is the number of links a node has, represented as a proportion of the greatest number of links within the network (Equation 1). Closeness centrality is the simplest centrality measure that considers movement between nodes (Bavelas, 1950). It represents the minimum number of steps between the node in question and all other nodes (Equation 2). This distance is related to the generated graph, and not a physical distance, and is also represented as a proportion of the greatest closeness value.

Degree centrality:

(1) $$\text{Degree}\;\left(v\right)=\;\frac{n_{v}}{n_{j}}\;$$

where *v* = node in question, *n* = number of links, *j* = node with the greatest number of links.

Closeness centrality:

(2) $$\text{Closeness}\;\left(v\right)=\;\frac{1}{\sum_{i\neq v}d_{\mathrm{vi}}}\;\;\;$$

where *d* = the minimum number of steps between two nodes, *v* = node in question, and *i* = next node.

If a tagged swift had multiple detections at one roost, these were pooled into a single detection to reduce the likelihood of pseudoreplication. After determining the degree and closeness centrality of each roost, we removed those with the greatest values and used a Wilcoxon‐Pratt signed‐rank test to determine the significance of their loss over the network. We generated network graphs to visualize the influence of roost removal on the structure of the overall network.

## 3 RESULTS

A total of 1,122 detections of 21 tagged swifts were recorded after data were filtered to exclude false positives (run lengths ≤ 3, Crewe et al., 2018) and limited to evening and morning (18:00–10:00). We detected tagged birds at a total of 12 Motus towers throughout southern Nova Scotia within the evening and morning time restriction (Figure 1). Two of these towers were <10 km, and one was <20 km of a single roost (Weymouth) and so were pooled to represent data from that roost. Three Motus towers along the eastern shore detected tagged swifts with no known roosts nearby. These sites were included in the network as potential roosts. Finally, there were two Motus towers >25 km from any known roosts along the western shore. These detections were primarily between 1 hr00–05 hr00 (Marshalltown) and 20 hr00–08 hr00 (Upper Clements), indicating the tagged swifts (eight total) were in the area overnight. As such, these sites were also retained as unknown roost sites, resulting in 10 total roosts included in the network analysis (Figure 1). Of the 21 birds that were detected, five (24%) used a single roost, five (24%) used two, nine (43%) used three, and two (10%) used four roosts. In total, 76% of tagged swifts did not show roost site fidelity, using more than one roost throughout the breeding season. Of those that did show roost site fidelity, it is important to note their breeding status could not be confirmed.

All centrality measures identified Caledonia as the most important roost site (Table 2), despite removing all initial detections at the site from analyses (Table 1). Degree centrality found Upper Clements and Bridgetown to be the second and third most important roosts, while closeness centrality identified Upper Clements and Marshalltown. Based on these results, we individually removed the Caledonia, Bridgetown, Marshalltown, and Upper Clements roosts from the network and recalculated the centrality measures to determine if the network was significantly altered.

**TABLE 2:** Centrality measures of the chimney swift (*Chaetura pelagica*) roost network in southern Nova Scotia without the presence of roosts with the greatest centrality measures

Roost	All sites		No Bridgetown		No Caledonia		No Marshalltown		No Upper Clements	
	Degree	Closeness	Degree	Closeness	Degree	Closeness	Degree	Closeness	Degree	Closeness
Blandford	0.180	0.056	0.222	0.045	0.200	0.042	0.182	0.063	0.128	0.040
Bridgetown	0.730	0.059	NA	NA	1.000	0.045	0.727	0.067	1.000	0.048
Caledonia	1.000	0.077	0.889	0.053	NA	NA	1.000	0.091	0.875	0.053
Jordan Bay	0.090	0.040	0.111	0.038	0.200	0.038	0.091	0.043	0.125	0.037
Liverpool	0.090	0.042	0.111	0.038	0.200	0.042	0.091	0.045	0.000	0.014
Marshalltown	0.450	0.063	0.556	0.050	1.000	0.071	NA	NA	0.250	0.043
Middleton	0.360	0.040	0.000	0.014	0.500	0.034	0.364	0.045	0.500	0.037
Upper Clements	0.820	0.063	1.000	0.050	1.000	0.059	0.545	0.067	NA	NA
Weymouth	0.360	0.059	0.444	0.048	0.400	0.053	0.273	0.063	0.500	0.048
Wolfville	0.270	0.059	0.222	0.043	0.400	0.059	0.182	0.063	0.375	0.047
		*z*	−0.296	**2.668**	**−2.668**	1.792	0.831	−1.602	−0.534	**2.669**
		*p*	0.767	**0.008**	**0.008**	0.073	0.406	0.109	0.594	**0.008**

Note

All loops have been removed, and the total sample is 1,122 detections of 21 tagged birds throughout June–September 2018 and 2019. Bold text denotes statistical significance (roosts with the greatest degree and/or closeness centrality).

Different centrality measures showed varying influence of roost removal from the network (Table 2). Upper Clements and Bridgetown were both found to significantly alter the network to the same extent based on closeness centrality (*z* = 2.668, *p* = .008). Caledonia was only significant when considering degree centrality (*z* = −2.668, *p* = .008). Neither centrality measure showed Marshalltown as having significant influence if removed, so network graphs were not constructed without Marshalltown.

**FIGURE 2:**
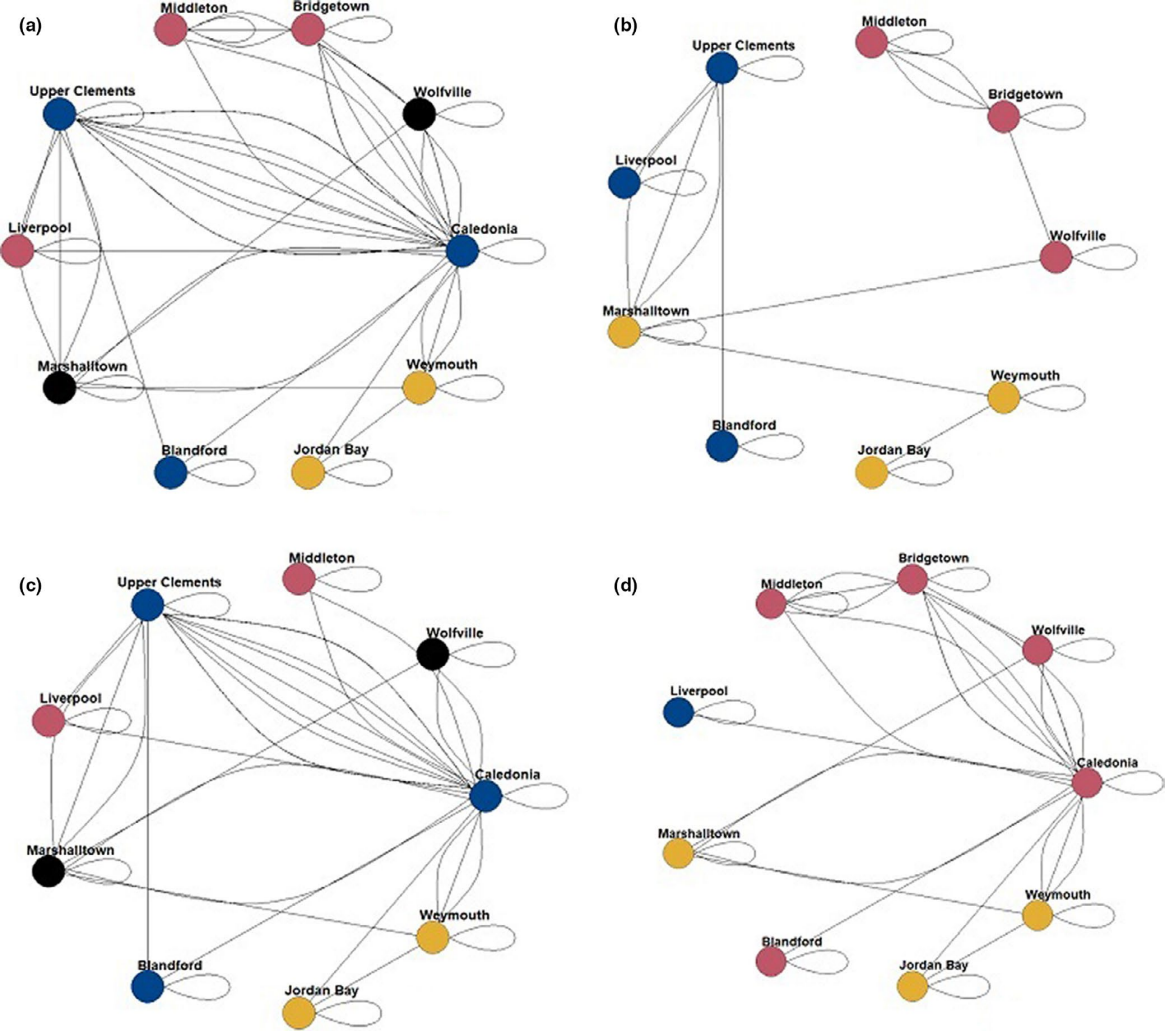
Chimney swift (*Chaetura pelagica*) community structure and roost networks determined through social network analysis (a). Observations consisted of 1,122 observations of 21 ratio tagged chimney swifts over June – 1 September 2018 and 2019. Node color is indicative of community structure. Roosts with the greatest degree (b: Caledonia) and closeness (c: Bridgetown, d: Upper Clements) centralities were individually removed to examine effect on community and network structure

When plotting the network and community structures with all sites (Figure 2a), without Caledonia (Figure 2b), Upper Clements (Figure 2c), or Bridgetown (Figure 2d), we can observe both the communities and the network structure without these roosts. There is minimal difference between the full network and that without the Bridgetown roost. However, without the Caledonia or Upper Clements roosts the network became simple. Community structure within each network is also altered, resulting in an isolated roost site without Upper Clements and Bridgetown. Bridgetown was the only roost whose loss does not diminish the total number of communities.

## 4 DISCUSSION

Our results show that SNA can be used to determine the existence and extent of networks of important ecological features. Our study is the first to track the movement of individual swifts between and among roosts throughout the breeding period. By using SNA as we did, not only could we show that there is roost switching by swifts, but that the extent of movement forms a complex network over a large landscape. We found that 76% of swifts used more than one roost site throughout the breeding season, which has never been documented. This indicates the importance of managing roosts as a network, as many swifts move between roosts over a large spatial scale. Though this could have been shown using mapping alone, SNA provides a quantifiable measure of the relative importance of each roost to maintaining connectivity within the network. This new insight into the ecology of chimney swifts could have management implications in terms of a species recovery plan for this species at risk. These results show that SNA can provide insight of how features are connected, the complexity of systems, and how to focus conservation and management efforts.

Social network analysis could be a valuable tool for identifying and conserving important roost sites for chimney swifts. This is crucial from a conservation perspective, due to the limiting nature of roosting chimneys currently available across the landscape and the high risk of their loss due to human disuse. With limited natural roosting sites available in the form of large hollow trees, and a preference for chimneys (Graves, 2004; Steeves et al., 2014), the conservation of chimneys is key to the persistence of this species as they fulfill a vital ecological requirement for swifts by providing an area to rest (Steeves et al., 2014), conserve energy (Du Plessis & Williams, 1994; Lubbe et al., 2018), and receive protection from the environment (Combrink et al., 2017; Walsberg, 1986). With few of these chimneys remaining, the loss of more could result in increased energy expenditure as swifts are forced to move further to find suitable roosting sites. SNA is rarely used in relation to species at risk (Webber & Vander Wal, 2019), and our work highlights the applicability of SNA in this context.

When considering the spatial position of each roost and their division into communities (Figure 1), the importance of the Caledonia site is clear. Caledonia is the only known roosting site in the interior of the southern portion of Nova Scotia and likely provides an important stopover site for swifts moving between the eastern and western shores. The return of individuals to the Caledonia roost after moving to the western shore could also be indicative of habitat quality, as the site is surrounded by protected areas (including Kejimkujik National Park), forests, wetlands, and lakes. This habitat may provide higher quality or a greater abundance of prey items than coastal sites, which have been found to have a lower abundance of aerial insects (Russell & Wilson, 2001). This could indicate the importance of natural areas for the species while foraging.

At a broader scale, these results show the applicability of SNA for understanding social interactions with key ecological features. These results also show promise for use in the identification of key roosting sites for socially roosting bats and birds and can be applied to a wider range of species. This approach may be especially applicable to studies of migratory stopover sites and how they form interactive networks over a large landscape. The ability to quantify the importance of individual features and examine the influence of removal on the theoretical network structure opens the possibility of targeted conservation planning from the scale of a single key roost site to the larger scale of a habitat patch in a fragmented landscape.

Many complex network structures likely exist in nature and using SNA to evaluate their interactions, extent, and effect of loss could be valuable in future conservation efforts. With advances in technology that allow for greater collection of movement data, SNA in the context of this study can provide a unique and useful method of evaluating and understanding species interaction with important landscape features. SNA will be important as further advances provide finer‐scale data.

The most significant limitation in this study is that all swifts were tagged at a single roost, the Caledonia site. If swifts did show roost fidelity, this would bias the results by increasing the relative importance of the Caledonia roost. To minimize this risk, only birds that left and then returned to Caledonia were considered a link for the roost (Table 1). Despite being the sole tagging site, the Caledonia roost is still a key roost within the network. It is the only known roost in the interior of the province and acts as a stopover for swifts moving between coasts.

Another limitation of this study is that each season used a different method of detection (handheld vs. automated), with each method having its own bias. With handheld telemetry, it is difficult to locate individuals as mobile as swifts, resulting in fewer overall detections, though interpretations of detections are more intuitive and straightforward. With the use of automated telemetry towers, we gained a greater number of location points but were limited by the tower locations. As such, some known roost sites were not sampled well due to the lack of a nearby Motus tower, while many detections were in areas with no known roosting sites. This last point may indicate the presence of an unknown roost site in these locations and should be investigated. Alternately these detections may represent swifts foraging in the area and not actually using the roost. We attempted to account for this possibility by limiting detections to the evening and early morning when swifts are typically found near their roost. To further this line of research, automated telemetry towers should be placed at each roost within the study site to increase detection, though this adds bias to known sites. Additionally, swifts should be tagged at multiple roosts to reduce the bias associated with single‐site capture and tagging.

Future research should address the limitations present in this study. Tagging should occur at multiple sites and all sites of interest should have a dedicated automated telemetry tower. This would eliminate the biases and limitations of this current study and provide more in‐depth results that allow conservation decision making. Future studies should also include the ability of SNA to identify roost networks in other areas and should also aim to monitor more individuals over several years to determine the true extent and variability of this network. The influence of covariates should also be addressed, such as influence of weather variables on movement between sites. This study highlights the use of SNA for identifying networks and movement between nodes, but the mechanisms driving these movements are currently unknown.

## CONFLICT OF INTEREST

None declared.

## AUTHOR CONTRIBUTION

**Courtney E. le Roux:** Conceptualization (equal); Data curation (lead); Formal analysis (lead); Investigation (lead); Methodology (equal); Visualization (lead); Writing‐original draft (lead). **Joseph J. Nocera:** Conceptualization (equal); Data curation (supporting); Formal analysis (supporting); Funding acquisition (lead); Investigation (lead); Methodology (equal); Project administration (lead); Resources (lead); Supervision (lead); Writing‐original draft (supporting); Writing‐review & editing (lead).

## PERMITS

Approval of handling protocols was given by the University of New Brunswick's Animal care Committee (Animal Use Protocol no. 18103). Conditions for banding and radio‐tagging swifts were applied to JJN’s master banding permit #10801 (Canadian Wildlife Service).

## Data Availability

The dataset used in this article is archived at Dryad (https://doi.org/10.5061/dryad.m63xsj415).
